# Type 2 diabetes mellitus in acute myocardial infarction: a persistent significant burden on long-term mortality

**DOI:** 10.3389/fcvm.2024.1401569

**Published:** 2024-06-12

**Authors:** Frédéric Bouisset, Vincent Bataille, François Schiele, Etienne Puymirat, Antoine Fayol, Tabassome Simon, Nicolas Danchin, Jean Ferrières

**Affiliations:** ^1^ Department of Cardiology, Toulouse Rangueil University Hospital, INSERM UMR 1295, Toulouse, France; ^2^ Association Pour la Diffusion de la Médecine de Prévention (ADIMEP), Toulouse, France; ^3^ Department of Cardiology, University Hospital Jean Minjoz, Besançon, France; ^4^ Department of Cardiology, Assistance Publique-Hôpitaux de Paris (AP-HP), Hôpital Européen Georges Pompidou, Université Paris-Descartes, Paris, France; ^5^ Department of Clinical Pharmacology and Unité de Recherche Clinique (URCEST), AP-HP, Hôpital Saint Antoine, Université Pierre et Marie Curie (UPMC-Paris 06), Paris, France; ^6^ Department of Cardiology, Hôpital Saint Joseph, Paris, France

**Keywords:** acute myocardial infarction, acute coronary syndrome, percutaneous coronary intervention, diabetes, prognosis

## Abstract

**Objective:**

The long-term impact of type 2 diabetes mellitus (T2DM) after an acute myocardial infarction (AMI) has not been thoroughly investigated yet. This study aimed to assess the long-term impact of T2DM after AMI.

**Research design and methods:**

We analyzed the data of three nationwide observational studies from the French Registry of Acute ST-elevation and non-ST-elevation Myocardial Infarction (FAST-MI) program, conducted over a 1-month period in 2005, 2010, and 2015. Patients presenting T2DM were classified as diabetic, and patients presenting type 1 diabetes mellitus were excluded. We identified factors related to all-cause death at 1-year follow-up and divided 1,897 subjects into two groups, paired based on their estimated 1-year probability of death as determined by a logistic regression model.

**Results:**

A total of 9,181 AMI patients were included in the analysis, among them 2,038 (22.2%) had T2DM. Patients with diabetes were significantly older (68.2 ± 12.0 vs. 63.8 ± 14.4, *p* < 0.001) and had a higher prevalence of a prior history of percutaneous coronary intervention (PCI), coronary artery bypass grafting (CABG), or heart failure (22.5% vs. 13.0%, 7.1% vs. 3.1% and 6.7 vs. 3.8% respectively, *p* < 0.001 for all). Even after matching two groups of 1,897 patients based on propensity score for their 1-year probability of death, diabetes remained associated with long-term mortality, with an HR of 1.30, 95%CI (1.17–1.45), *p* < 0.001.

**Conclusions:**

T2DM *per se* has an adverse impact on long-term survival after myocardial infarction. Independently of the risk of short-term mortality, patients with diabetes who survived an AMI have a 30% higher risk of long-term mortality.

## Introduction

Diabetes is widely recognized as a significant factor greatly increasing the risk of developing atherosclerotic cardiovascular diseases, such as myocardial infarction (MI) (1, 2). Importantly, 25 years ago, Haffner et al. (3) reported that the risk of MI was the same for a patient with diabetes compared to a patient without diabetes but with a prior personal history of MI, which was confirmed by other studies later on (4).

It has also been demonstrated that patients with diabetes presenting with an MI have a worse prognosis both during index hospitalization and during follow-up (5–7), despite recent improvements in both diabetes and AMI management (8–11). However, patients with diabetes are usually at higher risk, not only regarding their initial clinical and paraclinical presentation but also regarding their comorbidities. Therefore, numerous confounders may affect the evaluation of the impact of diabetes on clinical outcomes. Moreover, in studies focusing on the effect of diabetes, follow-up duration is often limited to 1 year, and in many cases, potential confounders are not all considered to properly assess the effect of diabetes alone.

Therefore, the objective of this study was to evaluate the long-term impact of T2DM *per se*, on vital prognosis after a MI by pooling the data from three sequential nationwide French surveys conducted between 2005 and 2015.

### Research design and methods

#### Study population

Three nationwide French registries were conducted over a 1-month period, 5 years apart, over a 10-year period (2005–2015): FAST-MI (French Registry of Acute ST-Elevation or non-ST-elevation Myocardial Infarction) 2005 (NCT00673036) (12), FAST-MI 2010 (NCT01237418) (13), and FAST-MI 2015 (NCT02566200) (14) (Online Data Supplements). The methods used to conduct these registries were detailed previously (12–15). In summary, their primary objectives were to assess the characteristics, management, and outcomes of acute myocardial infarction (AMI) patients within routine clinical practice on a country-wide scale.

All three registries consecutively included patients with STEMI admitted to intensive cardiovascular care units (ICCUs) within 48 h of symptom onset, during a specified 1-month period (October–December 2005, 2010, and 2015). AMI was defined by increased levels of cardiac biomarkers (troponins, CK, or CK-MB) together with either compatible symptoms or ECG changes. Patients who died soon after admission and for whom cardiac markers were not measured were included if they had signs or symptoms associated with typical ST-segment changes. A total of 13,129 patients were included in the three surveys.

The study was conducted in accordance with guidelines on good clinical practice and French regulations. The 2005 registry was reviewed and approved by the Committee for the Protection of Human Subjects (CPP) in Biomedical Research of Saint Antoine University Hospital, Paris; the 2010 registry was reviewed and approved by the CPP of Saint Louis University Hospital, Paris; and the protocol of the 2015 registry was reviewed and approved by the CPP of Saint Louis University Hospital, Paris Ile de France IV. Data file collection and storage were approved by the Commission Nationale de l'Informatique et des Libertés. Written consent was obtained for all these surveys.

#### Patient selection

**Figure 1:**
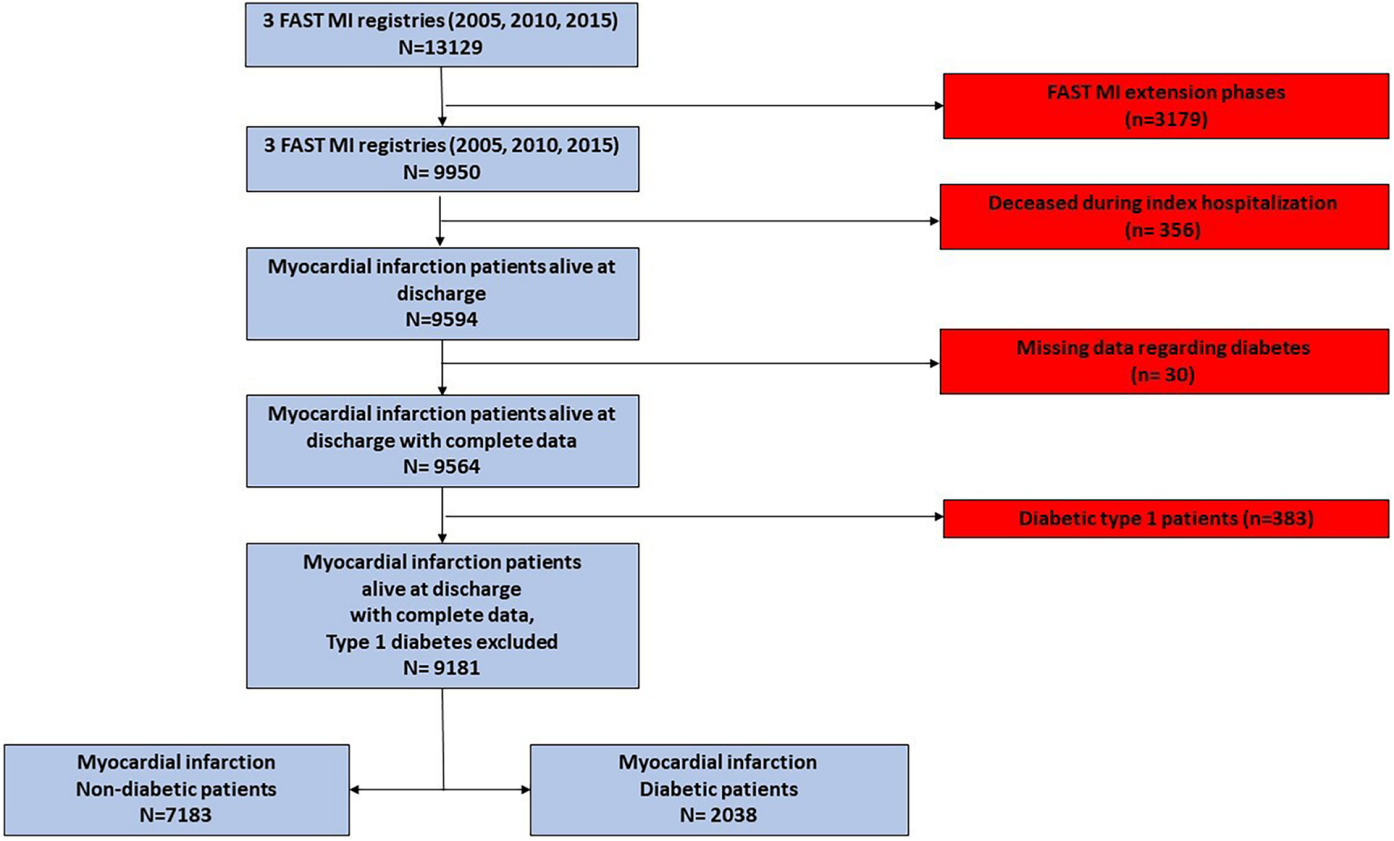
Study flowchart. The study population was derived from three French nationwide 1-month registries of myocardial infarction (FAST-MI registries) conducted in 2005, 2010, and 2015.

The patients included in the FAST-MI 2005 extension phases were excluded (*n* = 3179), as were those deceased within index hospitalization (*n* = 356), to exclude potential immortal time bias. After the exclusion of patients for whom data regarding diabetes status was missing (*n* = 30) and patients presenting a diabetes type 1 (*n* = 383), a total of 9,181 AMI patients (STEMI and non-STEMI) were assessed. A detailed flowchart is provided in Figure 1.

#### Data collection

Data on baseline characteristics, including demographics, medical history, and initial electrocardiogram (ECG), were collected as previously described (12–14, 16, 17). Information on the use and type of reperfusion therapy (primary PCI or fibrinolysis) in STEMI patients, the use of cardiac procedures [coronary angiography, PCI, intra-aortic balloon pump (IABP), and other cardiac devices], and mechanical ventilation were recorded over the entire hospital stay. Use of medications, administered in the pre-hospital setting, within the first 48 h and at-hospital discharge were collected. Additional variables such as previous PCI, coronary artery bypass graft surgery (CABG), chronic renal failure, laboratory data (C-reactive protein), or left ventricular ejection fraction were also recorded. Clinical complications at admission or during the initial hospital course and transfer to the general ICU were also recorded. Follow-up parameters, including death rate, recurrent AMI, stroke, all-cause death, all-cause hospitalization, cardiovascular hospitalization, hospitalization for heart failure, and bleeding were centralized at the French Society of Cardiology (SFC).

T2DM was defined by the presence of a personal medical history of diabetes mentioned in the medical record of the subject, inclusion of diabetes medications during the index hospitalization or at discharge (ATC A10), or an HBA1c level equal to or greater than 6.5%, as assessed during the index hospitalization. Patients treated with insulin alone were considered as having Type 1 diabetes and were excluded from this analysis.

#### Outcomes

Our primary outcome was all-cause mortality during follow-up.

Follow-up data were collected yearly by research technicians from the French SFC using the following sequential procedure:

(1) consulting the registry offices of the patients' birthplaces for death certificates;
(2) contacting the patients' general practitioners and/or cardiologists;
(3) contacting the patients or their relatives. In many instances, written communication was followed by telephone interviews with the patients or their families;
(4) consulting the French national database, which records all deaths occurring in the French population (RNIPP: Répertoire National d’Itentification des Personnes Physiques).

For each reported event leading to hospitalization or death, hospital discharge reports were sought and analyzed by at least one physician from the research team. All cases of cardiovascular events were centrally reviewed by at least one physician. Cases in which the final diagnosis appeared unclear or debatable were further reviewed by a three-member clinical events committee.

#### Statistics

Continuous data were expressed as mean ± standard deviation when following a normal distribution and as median (interquartile range) otherwise. Categorical data were displayed as counts and percentages. Group comparisons were conducted using Student's *t*-tests or Mann–Whitney non-parametric tests for continuous variables and using *χ*^2^ or Fisher exact tests for categorical variables.

### Mortality at 1 year and propensity score matching

Factors independently associated with 1-year mortality were identified using stepwise backward logistic regression. Apart from diabetes, all parameters associated with 1-year mortality with *p* < 0.2 in univariate analyses were included in the initial model. The predicted probability obtained from the final model was then used to perform a propensity score matching (1:1), allowing for comparison between long-term survival in diabetic and non-diabetic patients, after controlling for the effect of factors influencing short-term (1-year) mortality.

### T2DM and long-term survival

Survival was assessed in the more or less long term depending on the amount of follow-up available between the start date of each of the three studies (2005, 2010, 2015) and the time at which these analyses were carried out. In the propensity-matched cohort and therefore regardless of the probability of short-term death from the initial AMI, “residual” long-term risk associated with T2DM was assessed by computing the crude HR attributable to diabetes using a Cox regression model. Sensitivity analyses were performed stratified on the year of the registry and type of acute coronary syndrome (STEMI or non-STEMI).

Statistical significance was defined by *p* < 0.05 for all tests. All statistics were calculated using Stata Statistical Software [StataCorp (2021). Stata Statistical Software: Release 17. College Station, TX, USA]

## Results

### Study population

A total of 9,181 AMI patients with complete data were included in the three pooled FAST-MI registries (2,733 subjects in FAST-MI 2005, 2,874 in FAST-MI 2010, and 3,574 in FAST-MI 2015). Among them, 2,038 (22.2%) had T2DM.

### Patient presentation

Patients with T2DM were significantly older (68.2 ± 12.0 vs. 63.8 ± 14.4, *p* < 0.001) than patients without, with no difference among sexes (72.1% and 73.0% of men, respectively, in patients with T2DM and without, *p* = 0.42). Hypertension and dyslipidemia were more prevalent in the patients with T2DM (74.5% vs. 47% and 56.8% vs. 40.3% respectively, *p* < 0.001 for both). Conversely, a family history of CHD was less prevalent in patients with T2DM (21.6% vs. 27.4% *p* < 0.001). Among patients with T2DM, prior history of PCI, CABG, or heart failure was significantly more frequent than in patients without (22.5% vs. 13.0%, 7.1% vs. 3.1%, and 6.7 vs. 3.8%, respectively, *p* < 0.001 for all). Moreover, a past medical history of AF/flutter, stroke/TIA, PAD, and chronic kidney disease was also more prevalent in patients with T2DM (8.1% vs. 5.2%, 7.7% vs. 4.8%, 11% vs. 5.8%, and 6.4 vs. 2.8%, respectively, *p* < 0.001 for all).

At admission, obese patients (IMC ≥ 30 kg/m^2^) were twice as common in the group with T2DM (35.7 vs. 17.4%, *p* < 0.001). ST-elevation or new-onset LBBB was less common in patients with diabetes (45.1% vs. 56.1%, *p* < 0.001); however, cardiogenic shock (i.e., Killip IV) at presentation was more likely to be present in the group with T2DM (1.5% vs. 0.8%, *p* < 0.001). A description of patient characteristics according to their diabetes status is shown in Table 1, and a comparison of T2DM and non-T2DM patient characteristics according to their year of admission is presented in Supplementary Table S1.

**Table 1:** Comparison of characteristics and management in patients with diabetes and patients without.

	All *n* = 9,181	Without T2DM *n* = 7,143	With T2DM *n* = 2,038	*p*
Demographic and social data				
Male gender	6,682 (72.8)	5,213 (73)	1,469 (72.1)	0.421
Age (years)^a^	64.8 ± 14.0	63.8 ± 14.4	68.2 ± 12.0	<0.001
*n*	9,181	7,143	2,038	
Current situation				<0.001
Active	1,886 (33.0)	1,651 (37.1)	235 (18.7)	
Unemployed	217 (3.8)	168 (3.8)	49 (3.9)	
Disability/disease	206 (3.6)	157 (3.5)	49 (3.9)	
Retired	3,400 (59.6)	2,473 (55.6)	927 (73.6)	
Lives alone	1,408 (22.3)	1,090 (22.2)	318 (22.8)	0.673
Cardiovascular risk factors and medical history				
Hypertension	4,870 (53.1)	3,354 (47)	1,516 (74.5)	<0.001
Dyslipidemia	4,022 (43.9)	2,871 (40.3)	1,151 (56.8)	<0.001
Smoking				<0.001
No	3,528 (39.3)	2,658 (38.0)	870 (43.9)	
Former smoker	2,251 (25.1)	1,652 (23.6)	599 (30.2)	
Active smoker	3,194 (35.6)	2,679 (38.3)	515 (26)	
Family history of CAD	2,265 (26.2)	1,861 (27.4)	404 (21.6)	<0.001
First cardiac event	6,885 (75.1)	5,569 (78.1)	1,316 (64.6)	<0.001
Previous heart failure	408 (4.5)	273 (3.8)	135 (6.7)	<0.001
Previous PCI	1,381 (15.1)	924 (13.0)	457 (22.5)	<0.001
Previous CABG	360 (3.9)	217 (3.1)	143 (7.1)	<0.001
Previous (non-coronary) cardiac surgery	152 (1.7)	98 (1.4)	54 (2.7)	<0.001
Previous AF or flutter	376 (5.9)	261 (5.2)	115 (8.1)	<0.001
Previous stroke/TIA	500 (5.5)	343 (4.8)	157 (7.7)	<0.001
PAD	638 (7.0)	414 (5.8)	224 (11)	<0.001
Chronic renal failure	331 (3.6)	202 (2.8)	129 (6.4)	<0.001
Dialysis	62 (0.7)	39 (0.5)	23 (1.1)	0.005
Other life-threatening diseases	1,754 (19.2)	1,290 (18.1)	464 (22.8)	<0.001
Initial symptoms				
Asymptomatic	106 (1.2)	69 (1)	37 (1.8)	0.002
Pre-hospitalization heart failure	739 (8.4)	460 (6.7)	279 (14.2)	< 0.001
Syncope	364 (4.1)	301 (4.4)	63 (3.2)	0.024
Cardiac arrest	113 (1.3)	96 (1.4)	17 (0.9)	0.066
Typical chest pain	7,428 (82.0)	5,846 (82.7)	1,582 (79.1)	<0.001
Clinical characteristics at entry				
BMI (kg/m^2^)	26.9 ± 4.6	26.3 ± 4.3	28.9 ± 5.1	<0.001
*n*	8,613	6,704	1,909	
Obesity (BMI ≥ 30 kg/m^2^)	1,847 (21.4)	1,166 (17.4)	681 (35.7)	<0.001
Heart rate (bpm)	77.7 ± 18.4	76.9 ± 18.2	80.5 ± 18.6	<0.001
*n*	8,877	6,894	1,983	
Systolic BP (mmHg)	138 ± 27	137 ± 26	143 ± 27	<0.001
*n*	8,908	6,928	1,980	
Diastolic BP (mmHg)	79 ± 16	79 ± 16	79 ± 16	0.695
*n*	8,892	6,916	1,976	
Killip class at entry				<0.001
I	7,575 (86.4)	6,049 (88.5)	1,526 (78.9)	
II	736 (8.4)	515 (7.5)	221 (11.4)	
III	377 (4.3)	220 (3.2)	157 (8.1)	
IV	81 (0.9)	52 (0.8	29 (1.5)	
Max Killip class during hospitalization				<0.001
I	7,255 (83.8)	5,807 (85.9)	1,448 (76.3)	
II	774 (8.9)	547 (8.1)	227 (12)	
III	455 (5.3)	287 (4.2)	168 (8.9)	
IV	174 (2.0)	120 (1.8)	54 (2.8)	
STEMI or LBBB at entry	4,923 (53.6)	4,004 (56.1)	919 (45.1)	<0.001
Biological data upon admission				
Triglycerides (mg/dl)^b^	81 (50–129)	79 (49–124)	92 (56–146]	<0.001
*n*	7,070	5,500	1,570	
LDL cholesterol (mg/dl)	127 ± 49	131 ± 048	114 ± 50	<0.001
*n*	6,587	5,141	1,446	
HDL cholesterol (mg/dl)	47 ± 17	47 ± 17	44 ± 16	<0.001
*n*	6,796	5,289	1,507	
Glycemia (mg/dl)^b^	120 (100–154)	120 (100–140)	170 (130–230)	<0.001
*n*	7,978	6,171	1,807	
Hemoglobin (g/dl)	14.0 ± 1.9	14.1 ± 1.8	13.7 ± 2.0	<0.001
*n*	8,899	6,926	1,973	
CRP (UI/L)^b^	5.0 (2.9–13.0)	5 (2.5–11.5)	6 (3.0–19.0)	<0.001
*n*	6,678	5,142	1,536	
CKD-EPI creatinine clearance (ml/min/1,73 m^2^)				<0.001
≥90	2,864 (32.1)	2,361 (34.0)	503 (25.6)	
60–89	3,826 (42.9)	3,027 (43.6)	799 (40.6)	
30–59	1,714 (19.2)	1,207 (17.4)	507 (25.8)	
15–29	207 (2.3)	125 (1.8)	82 (4.2)	
<15 or dialysis	301 (3.4)	225 (3.2)	76 (3.9)	
HbA1c (%)			7 (6.5–7.9)	NA
*n*			1,306	
Coronarography—reperfusion therapy				
Coronary angiography performed	8,638 (94.1)	6,751 (94.5)	1,887 (92.6)	0.001
Coronary disease extension (when CAD was detected by angiography)^c^				<0.001
1-vessel disease	3,841 (47.4)	3,166 (50.2)	675 (37.7)	
2-vessel disease	2,554 (31.5)	1,937 (30.7)	617 (34.4)	
3-vessel disease	1,708 (21.1)	1,207 (19.1)	501 (27.9)	
Any PCI attempt during initial hospitalization	6,922 (75.4)	5,448 (76.3)	1,474 (72.4)	<0.001
Any CABG during initial hospitalization	293 (3.2)	204 (2.9)	89 (4.4)	0.001
Initial LVEF (%)	52.1 ± 11.6	52.6 ± 11.4	50.2 ± 12.2	<0.001
*n*	6,864	5,341	1,523	
Characteristics at discharge				
STEMI (discharge diagnosis)	4,835 (52.7)	3,949 (55.3)	886 (43.5)	<0.001
LVEF at discharge				<0.001
≥40%	5,052 (83.3)	3,981 (84.6)	1,071 (79)	
<40%	1,011 (16.7)	727 (15.4)	284 (21)	
LVEF at discharge (%)	52.3 ± 11	52.7 ± 10.8	51 ± 11.4	<0.001
* n*	6,063	4,708	1,355	
GRACE score discharge	111 ± 31	108 ± 31	119 ± 29	<0.001
*n*	8,498	6,626	1,872	
Treatment at discharge				
Beta-blocker at discharge	7,069 (77.0)	5,493 (76.9)	1,576 (77.3)	0.684
Statin at discharge	7,662 (83.5)	5,977 (83.7)	1,685 (82.7)	0.285
ACEI/ARB at discharge	6,238 (67.9)	4,777 (66.9)	1,461 (71.7)	<0.001
Anti-platelet agent at discharge	8,380 (91.3)	6,516 (91.2)	1,864 (91.5)	0.735

CAD, coronary artery disease; PAD, peripheral arterial disease; BP, blood pressure; LDL, low-density lipoprotein; HDL, high-density lipoprotein; CRP, C-reactive protein; CKD-EPI, chronic kidney disease—epidemiology collaboration; PCI, percutaneous coronary intervention; CABG, coronary artery bypass grafting; LVEF, left ventricular ejection fraction; STEMI, ST-elevation myocardial infarction; ACEI, angiotensin-converting enzyme inhibitor; ARB, angiotensin receptor blockers.

^a^ Mean ± SD.

^b^ Median (Interquartile range).

^c^ Left main lesion classified as a two-vessel disease.

### Patient management

Patients with T2DM less frequently underwent coronary angiographies (92.6% vs. 94.5 *p* = 0.001). As a result, these patients less frequently underwent PCI attempts (72.4% vs. 76.3% *p* < 0.001). Among patients presenting CAD at angiography, the severity of coronary artery disease was more pronounced in the group with T2DM in which 27.9% presented a three-vessel disease vs. 19.1% in the group without T2DM (*p* < 0.001). Consequently, patients with T2DM had more important myocardial damage with 21% of them leaving the hospital with an LVEF < 40% vs. 15.4% in the group without T2DM.

At discharge, no difference was observed regarding the prescription of antiplatelets, beta-blockers, and statins. ACE/ARB was significantly more prescribed in patients with T2DM (71.7% vs. 66.9% *p* < 0.001). The detailed management of patients according to their T2DM status is shown in Table 1.

### Diabetes medications

At admission, 60.6% of patients with known diabetes were taking antidiabetes therapies, meaning that 39.4% of them were treated by diet only. In addition, 39.9% of patients with diabetes were taking a biguanide, and 10.9% were under insulin therapy. At discharge, 64.6% of patients with diabetes were taking any antidiabetes therapy. The rate of biguanide prescription at discharge decreased to 29.6% whereas, in the meantime, the rate of insulin prescriptions increased to 23.3%. The detailed treatment of diabetes at admission and discharge is shown in Table 2, and the detailed treatment of diabetes according to the year of inclusion is presented in Supplementary Table S2. Between 2005 and 2015, biguanides remained the more frequent antidiabetes treatment at presentation.

**Table 2:** T2DM treatment at admission and discharge.

	Patients with diabetes *n* = 2,038	
	*n*	%
At the time of admission		
Any medical treatment for diabetes mellitus	1,235	60.6
Insulin treatment	222	10.9
Biguanide	813	39.9
Sulfonylurea	576	28.3
Alpha-glucosidase inhibitor	92	4.5
DPP4 inhibitor	161	7.9
Glucagon-like peptide-1 receptor agonists	19	0.9
Another oral antidiabetic agent	146	7.2
Medical treatment of T2DM at discharge		
Medical treatment for diabetes mellitus	1,317	64.6
Insulin treatment	474	23.3
Biguanide	604	29.6
Sulfonylurea	482	23.7
Alpha-glucosidase. inhibitor	50	2.5
DPP4 inhibitor	175	8.6
Glucagon-like peptide-1 receptor agonists	17	0.8
Another oral antidiabetic agent	133	6.5

DPP4, dipeptidyl peptidase-4.

### Impact of diabetes on the prognosis

During hospitalization and after adjustment for year of inclusion and age, the occurrence of complications did not differ between patients with or without T2DM except for blood transfusion, which remained rare in both groups but more frequent among patients with diabetes (4.3% vs. 2.7%, *p* = 0.033) and LVEF < 40%, also more frequent in the group with T2DM (21 vs. 15.4%, *p* < 0.001). However, during follow-up, all clinical events (recurrent MI, stroke, death, hospitalization for heart failure, other cardiovascular reasons, or any other reasons) were significantly more frequent among patients with diabetes. Clinical outcomes during hospitalization and follow-up are summarized in Table 3.

**Table 3:** In-hospital and long-term clinical outcomes according to T2DM status.

	All *n* = 9,181	Without T2DM *n* = 7,143	With T2DM *n* = 2,038	*p*	Adjusted p1^a^	adjusted p2^b^
In-hospital complications. n/total *n* (%)						
Recurrent MI	73/9,173 (0.8)	58/7,137 (0.8)	15/2,036 (0.7)	0.734	0.742	0.575
Stroke	39/9,166 (0.4)	32/7,133 (0.5)	7/2,033 (0.3)	0.524	0.525	0.522
TIMI major bleeding	57/9,169 (0.6)	43/7,134 (0.6)	14/2,035 (0.7)	0.666	0.702	0.835
TIMI minor bleeding	55/9,167 (0.6)	43/7,131 (0.6)	12/2,036 (0.6)	0.944	0.939	0.479
Blood transfusion	283/9,180 (3.1)	195/7,143 (2.7)	88/2,037 (4.3)	<0.001	<0.001	0.033
Atrial fibrillation	459/9,167 (5.0)	326/7,132 (4.6)	133/2,035 (6.5)	<0.001	<0.001	0.108
Ventricular fibrillation	146/9,164 (1.6)	122/7,130 (1.7)	24/2,034 (1.2)	0.093	0.093	0.195
LVEF ≤ 40%	1,011/6,063 (16.7)	727/4,708 (15.4)	284/1,355 (21.0)	<0.001	<0.001	0.001
30-day death	72/9,181 (0.8)	48/7,143 (0.7)	24/2,038 (1.2)	0.022	0.026	0.210
Complications during follow-up^c^						
Recurrent MI	7.3 (6.8–8.1)	6.7 (6.0–7.6)	9.5 (7.7–11.7)	0.011	0.013	0.017
Stroke	3.8 (3.3–4.4)	3.4 (2.8–4.0)	5.8 (4.4–7.5)	0.001	0.001	0.007
All-cause death	42 (40–43)	36 (34–38)	64 (60–69)	<0.001	<0.001	<0.001
All-cause hospitalization	166 (161–170)	155 (150–160)	211 (198–223)	<0.001	<0.001	<0.001
Cardiovascular hospitalization	88 (85–91)	82 (78–85)	116 (108–125)	<0.001	<0.001	<0.001
Hospitalization for heart failure	10.9 (10.0–11.9)	8.8 (7.8–9.8)	19.8 (17.1–22.9)	<0.001	<0.001	<0.001

MI, myocardial infarction; LVEF, left ventricular ejection fraction.

^a^ Adjusted for FAST-MI registry (2005. 2010 or 2015).

^b^ Adjusted for FAST-MI registry (2005. 2010 or 2015) and age.

^c^ Expressed as the number of events for 1,000 person-years (95% CI).

Factors associated with the occurrence of death at 1 year are presented with crude and adjusted for sex, sex, and year of inclusion OR in Supplementary Table S3. Supplementary Table S4 shows a multivariate analysis of factors independently related to the occurrence of death at 1-year follow-up (Hosmer and Lemeshow goodness of fit *p* = 0.51). From this multivariate analysis, a propensity score matching was done, allowing the constitution of two groups of 1,897 individuals, paired on their 1-year probability of death estimated by the logistic regression model.

**Figure 2:**
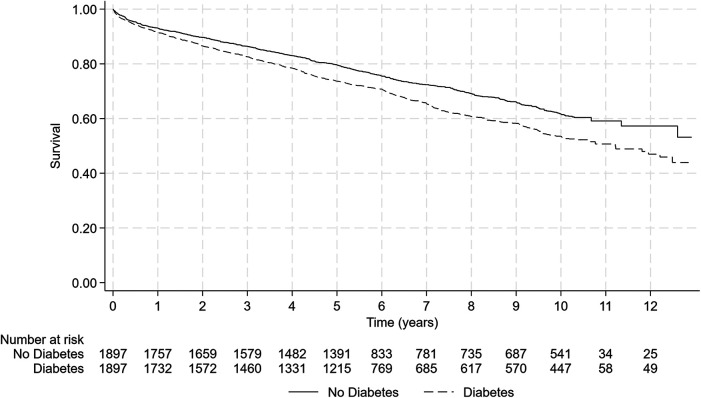
Mortality comparison according to T2DM status in matched populations by propensity score. The presence of T2DM is associated with a significant increase in mortality rate during follow-up compared to non-diabetic patients [log-rank test, *p* < 0.001—HR = 1.30 95%CI (1.17–1.45) *p* < 0.001].

Figure 2 presents the Kaplan–Meier mortality curves according to T2DM status in the two matched groups. In this analysis, diabetes was independently related to the occurrence of death during follow-up with an HR of 1.30 [95%CI (1.17–1.45), *p* < 0.001], meaning that in patients who survived an AMI and ignoring the factors impacting the risk of short-term (1 year) death, the long-term risk of death was 30% higher in patients with T2DM than in patients without.

## Discussion

This report investigates the impact of T2DM on long-term survival in patients presenting an AMI by pooling the data from three sequential nationwide French surveys conducted between 2005 and 2015. In our study including 9,181 patients, 2,038 (22.2%) had T2DM. After propensity score matching, diabetes was found related to the occurrence of death during long-term follow-up with an HR of 1.30 (95%CI (1.17–1.45), *p* < 0.001]. To the best of our knowledge, this study is the first to approach the “residual” impact of diabetes on long-term mortality risk in patients who suffered an AMI, considering the risk of death in the short term from the immediate consequences of this AMI.

In the present study, we aimed to quantify the impact of T2DM itself by using a propensity-matched analysis. It should be underlined that 93% of the patients with T2DM included could be matched to a patient without T2DM (2,038 patients with diabetes included in the study and 1,897 matched in the propensity analysis). This reinforces the reliability of our results. Potential confounding factors were considered with propensity matching, and we observed that the long-term impact of diabetes decreases from 1.76, 95% CI (1.62–1.92), *p* < 0.001 [crude HR for the long-term risk of death in 9,181 patients (Figure 1) alive at discharge (data not shown)] to 1.30, 95% CI (1.17–1.45), *p* < 0.001. This 30% increase in mortality corresponds to the residual burden of metabolic risk in T2DM. However, it should be underlined that recent studies have shown a significant reduction in diabetes complication rates (18), which suggests a global improvement in T2DM prognosis. Moreover, it should be emphasized that this increase in mortality remains after a complete adjustment of comorbidities. In the large UK AMI registry (19), MINAP, after cumulative adjustment for comorbidity, risk factors, and cardiovascular treatments, in ST-elevation MI, T2DM remained significantly associated with substantial excess mortality (excess mortality rate ratio, 95% CI), 1.56 (1.49–1.63). This suggests that additional factors are at play, such as medications during follow-up, drug adherence, and more rapid accumulation of microvascular complications in patients with T2DM (19).

Subsequently, the question of determining the cause of this 30% residual higher risk remains to be addressed. Several hypotheses can be advanced. One is to consider the potential responsibility of T2DM-related microvascular damages that are probably not entirely captured by the comorbidities on which the model could be adjusted (20). Indeed, as an example, data related to the presence of proteinuria, which would reflect a form of renal microvascular disease before the occurrence of renal dysfunction, were not available. Moreover, coronary microvascular dysfunction—highly prevalent in the T2DM population (20)—is also a condition that is not properly captured in our dataset. Indeed, although this condition can now be diagnosed and classified (21, 22), it requires specific invasive coronary measurements that are not appropriate in the setting of MI. Previous studies have demonstrated that patients with T2DM have a lower coronary flow reserve (CFR) than patients without T2DM (23). CFR is the ratio of hyperemic flow divided by resting flow and reflects the capacity of coronary blood flow to increase to meet myocardial oxygen needs (24). In the absence of significant epicardial disease, the reduction in CFR translates into the impaired vasoreactivity of the microcirculation, or “coronary microvascular dysfunction.” It is not completely clear, however, whether this reduction in CFR in patients with T2DM is driven by a higher resting flow (the so-called functional coronary microvascular dysfunction) or a decreased hyperemic flow (the so-called structural coronary microvascular dysfunction), as contradictory data have been reported (25, 26). Some authors also suggested that those two endotypes could reflect different phases during the evolution of the same disease (27). However, irrespective of the mechanism of CFR reduction, it is demonstrated that this has a strong prognosis impact (28). This condition, highly prevalent in the T2DM population and not captured in our dataset, is thus likely to play a role in the 30% increase in mortality, and explain why the risk of heart failure in this population plateaued since 2013, according to a large Swedish registry recently published (29).

Thirty years ago, the concept of “metabolic memory” was introduced to explain a phenomenon where the long-term vascular benefits of a previous period of good glycemic control persist despite a return to worse metabolic control (30). The metabolic memory, or the legacy effect, corresponds to the ongoing vascular injury as a result of prior transient episodes of poor metabolic control. An innovative strategy to attenuate the burden of complications resulting from prior hyperglycemia is to target the metabolic pathways that promote hyperglycemia memory (31).

### Study limitations

This study has several limitations. First, the diagnosis of diabetes is self-reported by the sites; however, HbA1c and treatment of diabetes are also considered in our definition of T2DM, which counterbalances this limitation. Second, we do not know the T2DM duration before the occurrence of acute coronary syndrome, and we also do not have data on the management of T2DM after the acute episode. Third, we lack data about microvascular diseases related to T2DM, such as diabetic cardiomyopathy, presence and severity of proteinuria, and peripheral neuropathy.

## Conclusion

The presence of T2DM in patients surviving an AMI has, *per se*, a long-term pejorative effect on global survival. Further research is needed to understand the underlying mechanisms of this to develop specific therapeutic strategies to limit the burden of diabetes on the health system.

## Data Availability

The original contributions presented in the study are included in the article/Supplementary Material, further inquiries can be directed to the corresponding author.
