# Supplementary material for: Type 2 diabetes mellitus in acute myocardial infarction: a persistent significant burden on long-term mortality
Source: Front Cardiovasc Med. 2024 Jun 12;11:1401569. doi: 10.3389/fcvm.2024.1401569 (PMC11204119; doi:10.3389/fcvm.2024.1401569)

Supplementary materials to manuscript:

Type 2 diabetes in acute myocardial infarction: A persistent significant burden on long-term mortality

Frédéric Bouisset *et al*

1 – Expanded methods:

Participation in the study was offered to all institutions, including university teaching hospitals, general and regional hospitals, and private clinics that received AMI emergencies. The number of participating centers was 223 in 2005, 213 in 2010, and 204 in 2015, respectively representing 60%, 76% and 78% of all centers taking care of AMI patients in France. Physicians were instructed that the study should not affect clinical care or management.

Methodology of the 3 surveys.

FAST-MI 2005:

- Sponsor: French Society of Cardiology
- Principal investigators: N. Danchin, T. Simon
- Funding: Pfizer, Servier, and additional grant from the French National Health Insurance (CNAM-TS).
- The protocol was reviewed by the Committee for the Protection of Human Subjects in Biomedical Research of Saint Antoine University Hospital (Paris, France).
- Aim: to evaluate practices for AMI management in "real life" practice, and to measure their impact on the medium- and long-term outcomes of patients admitted to intensive care units for AMI over a one month period in France.
- Institutions: voluntary participation of any institution authorized to take care of AMI patients, i.e. university hospitals, general hospitals, private clinics, with or without catheterization laboratory.
- Patient population: consecutive adult patients admitted to the participating centers and meeting the following criteria:
    - Inclusion criteria:
        - Diagnosis of AMI on the basis of elevated CK-MB or troponin, in combination with:
            - Symptoms compatible with prolonged myocardial ischemia

        - Or ECG changes compatible with myocardial ischemia: pathologic new Q waves or ST elevation, ST depression, or T wave inversion
      - Time from onset to admission < 48 hours
      - Patients who died very early after admission and for whom cardiac markers were not measured or not yet elevated were included if they had compatible signs or symptoms associated with typical ST changes.
  - Exclusion criteria:
      - Iatrogenic myocardial infarction
      - AMI diagnosis invalidated in favor of another diagnosis
- Period of inclusion: the study started in the participating centers between October 1st and November 15th 2005, inclusion for 31 consecutive days
- Electronic case record form with automated data queries, filled-in by dedicated research technicians traveling to each site at least once a week. Details (exact type and dose) of all medications prescribed at different time-points. Blood collection (DNA and serum) for core laboratory analysis, in the largest centers.

Ten-year follow-up centralized at the French Society of Cardiology, and performed by dedicated research technicians

FAST-MI 2010:

- Sponsor: French Society of Cardiology
- Principal investigators: N. Danchin, T. Simon
- Funding: MSD, the Daiichi-Sankyo/Eli-Lilly alliance, AstraZeneca, GSK, Novartis, Sanofi.
- The protocol was reviewed and approved by the Committee for the Protection of Human Subjects of Saint Louis University Hospital (Paris, France).
- Aim: to provide an extensive description of the population of patients admitted for AMI throughout the French territory, to determine whether differences in terms of population characteristics existed across regions, to assess the management of the patients suffering from AMI, and to determine the implementation of practice guidelines in a real world setting. Other objectives were to assess the correlations between management strategies and outcomes, to determine the correlations between genetic polymorphisms and morbimortality in relation with the effects of medications, and to determine relationships between biomarkers and morbimortality. Another objective was to enable historic comparisons with the previous French registries.
- Institutions: voluntary participation of any institution authorized to take care of AMI patients, i.e. university hospitals, general hospitals, private clinics, with or without catheterization laboratory.
- Patient population: consecutive adult patients admitted to the participating centers and meeting the following criteria:
    - Inclusion criteria:
        - Diagnosis of acute myocardial infarction on the basis of elevated CK-MB or troponin, in combination with:
            - Symptoms compatible with prolonged myocardial ischemia
            - Or ECG changes compatible with myocardial ischemia: pathologic new Q waves or ST elevation, ST depression, or T wave inversion
        - Time from onset to admission < 48 hours
        - Patients who died very early after admission and for whom cardiac markers were not measured or not yet elevated were included if they had compatible signs or symptoms associated with typical ST changes.
    - Exclusion criteria:

- Iatrogenic myocardial infarction
- AMI diagnosis invalidated in favor of another diagnosis

- Period of inclusion: the study started in the participating centers between October 1st and November 15th 2005, inclusion for 31 consecutive days
- Electronic case record form with automated data queries, filled-in by dedicated research technicians traveling to each site at least once a week. Details (exact type and dose) of all medications prescribed at different time-points. Blood collection (DNA and serum) for core laboratory analysis, in the largest centers.
- Ten-year follow-up centralized at the French Society of Cardiology, and performed by dedicated research technicians.

FAST-MI 2015:

- Sponsor: French Society of Cardiology
- Principal investigators: N. Danchin, T. Simon
- Funding: Amgen, AstraZeneca, Bayer, BMS, Boehringer-Ingelheim, the Daiichi-Sankyo-Eli-Lilly alliance, MSD, Sanofi.
- The protocol was reviewed and approved by the Committee for the Protection of Human Subjects of Saint Louis University Hospital Paris Ile de France IV (Paris, France).
- Aim: to provide a precise and extensive description of the population of patients admitted for AMI throughout the French metropolitan territory, and to determine whether regional differences existed in terms of patient population; to assess the management of the patients admitted to cardiology departments for AMI; to determine the actual implementation of practice guidelines in a real world setting; to assess the correlations between management strategies and in-hospital outcomes; to determine the impact of several genetic polymorphisms on morbidity-mortality and their interaction with the effect of medications; and, to determine the impact of biomarkers on morbidity-mortality after MI.
- Institutions: a list of all intensive cardiac care units (ICCU) authorized to receive ACS emergencies and admitting patients at the acute stage of MI was established at the beginning of 2015, and participation in the study was offered to all types of institutions (academic hospitals, general hospitals, army hospitals and private clinics). In all, 261 centers were listed, 215 of which initially accepted to participate in the study. Of those, 204 actively participated, and included at least one patient during the one-month study period. Participation rate was 78% and the centers were distributed across the whole country.
- Patient population: patients were recruited consecutively from ICCU/cardiology departments over a period of one month (from October, 5th 2015). Recruitment could be prolonged up to 2 months in the centers willing to do so. Inclusion and exclusion criteria were similar to previous registries:
  - Inclusion criteria:
    - Diagnosis of acute myocardial infarction on the basis of elevated CK-MB or troponin, in combination with:
      - Symptoms compatible with prolonged myocardial ischemia

        - Or ECG changes compatible with myocardial ischemia: pathologic new Q waves or ST elevation, ST depression, or T wave inversion
      - Time from onset to admission < 48 hours
      - Patients who died very early after admission and for whom cardiac markers were not measured or not yet elevated were included if they had compatible signs or symptoms associated with typical ST changes.
  - Exclusion criteria:
      - Iatrogenic myocardial infarction
      - AMI diagnosis invalidated in favor of another diagnosis
- Period of inclusion: the study started in the participating centers between October 1st and November 15th 2015, inclusion for 31 consecutive days
- Electronic case record form with automated data queries, filled-in by dedicated research technicians traveling to each site at least once a week. Details (exact type and dose) of all medications prescribed at different time-points. Blood collection (DNA and serum) for core laboratory analysis, in the largest centers; finally, some centers also collected stools for the purpose of studying intestinal flora.
- Ten-year follow-up centralized at the French Society of Cardiology, and performed by dedicated research technicians.

Data file collection and storage were approved by the French “Commission nationale de l’informatique et des libertés” for all registries.

2 – Supplementary tables

Supplementary table 1: Table 1: Comparison of T2DM and non- T2DM patients' characteristics and management according to year of inclusion

	FAST-MI 2005			FAST-MI 2010			FAST-MI 2015		
	**Without T2DM (n=2117)**	**With T2DM (n=616)**	**p**	**Without T2DM (n=2254)**	**With T2DM (n=620)**	**p**	**Without T2DM (n=2772)**	**With T2DM (n=802)**	**p**
**Demographic and social data**									
Male Sex	1489 (70.3)	429 (69.6)	0.741	1682 (74.6)	453 (73.1)	0.432	2042 (73.7)	587 (73.2)	0.789
Age (years) *	64.6 (14.9)	68.2 +/- 12.1	< 0.001	63.3 +/- 14.5	68.3 +/- 12.0	< 0.001	63.7 +/- 14.0	68 +/- 12.0	< 0.001
n	*2117*	*616*		*2254*	*620*		*2772*	*802*	
Current situation						< 0.001			< 0.001
Active				770 (36.8)	100 (17.5)		881 (37.4)	135 (19.6)	
Unemployed				89 (4.3)	25 (4.4)		79 (3.4)	24 (3.5)	
Disability / Disease				89 (4.3)	20 (3.5)		68 (2.9)	29 (4.2)	
Retired				1144 (54.7)	427 (74.7)		1329 (56.4)	500 (72.7)	
Lives alone				373 (19.5)	86 (17.0)	0.204	717 (30.3)	232 (33.2)	0.147
**Cardiovascular risk factors and medical history**									
Hypertension	1067 (50.4)	449 (72.9)	< 0.001	1039 (46.1)	458 (73.9)	< 0.001	1248 (45.1)	609 (76.2)	< 0.001
Dyslipidaemia	942 (44.6)	340 (55.2)	< 0.001	875 (38.8)	352 (56.8)	< 0.001	1054 (38.2)	459 (58)	< 0.001
Smoking			< 0.001			< 0.001			< 0.001
No	920 (43.3)	319 (51.8)		828 (37.2)	262 (43.4)		910 (34.3)	289 (37.8)	
Former smoker	459 (21.7)	160 (26)		529 (23.8)	195 (32.3)		664 (25.1)	244 (31.9)	
Active smoker	733 (34.7)	137 (22.2)		870 (39.1)	147 (24.3)		1076 (40.6)	231 (30.2)	
Family history of CHD	547 (26.6)	124 (20.9)	0.005	629 (28.7)	125 (21.1)	< 0.001	685 (27)	155 (22.7)	0.021
First cardiac event	1574 (74.5)	396 (64.3)	< 0.001	1796 (79.7)	399 (64.4)	< 0.001	2199 (79.5)	521 (65)	< 0.001

Previous Heart failure	97 (4.6)	30 (4.9)	0.766	71 (3.1)	40 (6.5)	< 0.001	105 (3.8)	65 (8.2)	< 0.001
Previous PCI	239 (11.3)	92 (15.0)	0.016	279 (12.4)	152 (24.6)	< 0.001	406 (14.8)	213 (26.7)	< 0.001
Previous CABG	80 (3.8)	52 (8.4)	< 0.001	77 (3.4)	36 (5.8)	0.006	60 (2.2)	55 (7.0)	< 0.001
Previous (non-coronary) cardiac surgery	33 (1.6)	18 (2.9)	0.028	18 (0.8)	10 (1.6)	0.068	47 (1.7)	26 (3.3)	0.006
Previous AF or Flutter				128 (5.7)	48 (7.7)	0.058	133 (4.8)	67 (8.4)	< 0.001
Previous Stroke/TIA	133 (6.3)	56 (9.1)	0.016	78 (3.5)	39 (6.3)	0.002	132 (4.8)	62 (7.8)	0.001
PAD	140 (6.6)	86 (14)	< 0.001	143 (6.3)	58 (9.4)	0.009	131 (4.7)	80 (10)	< 0.001
Chronic renal failure	72 (3.4)	37 (6)	0.004	55 (2.4)	31 (5)	< 0.001	75 (2.7)	61 (7.7)	< 0.001
Dialysis	14 (0.7)	5 (0.8)	0.693	10 (0.4)	7 (1.1)	0.049	15 (0.5)	11 (1.4)	0.014
Other life-threatening diseases	214 (10.1)	68 (11.1)	0.499	448 (19.9)	140 (22.6)	0.143	628 (22.8)	256 (32.1)	< 0.001
**Initial symptoms**									
Asymptomatic	14 (0.7)	8 (1.3)	0.119	10 (0.4)	8 (1.3)	0.018	45 (1.6)	21 (2.6)	0.066
Pre-hospitalization heart failure	176 (9.1)	93 (16.1)	< 0.001	189 (8.4)	109 (17.7)	< 0.001	95 (3.5)	77 (10)	< 0.001
Syncope	89 (4.6)	14 (2.5)	0.025	116 (5.2)	22 (3.5)	0.098	96 (3.6)	27 (3.5)	0.949
Cardiac arrest	33 (1.7)	5 (0.9)	0.153	22 (1)	4 (0.6)	0.44	41 (1.5)	8 (1)	0.317
Typical chest pain	1658 (79)	467 (77.7)	0.484	1931 (85.7)	498 (80.3)	0.001	2257 (83.1)	617 (79.3)	0.014
**Clinical characteristics at entry**									
BMI (kg/m²)*	26.2 +/- 4.3	28.8 +/- 5.2	< 0.001	26.3 +/- 4.3	29 +/- 4.9	< 0.001	26.4 +/- 4.4	28.8 +/- 5.2	< 0.001
Obesity (BMI ≥ 30 kg/m²)	320 (16.8)	186 (33)	< 0.001	358 (16.7)	222 (38.3)	< 0.001	488 (18.4)	273 (35.6)	< 0.001
Heart rate (bpm)*	77.9 +/- 20.0	82.3 +/- 19.7	< 0.001	75.7 +/- 16.7	79 +/- 16.4	< 0.001	77 +/- 17.8	80.3 +/- 19.2	< 0.001
n	*2108*	*613*		*2181*	*602*		*2605*	*768*	
Systolic BP (mmHg)*	138 +/- 27	144 +/- 29	< 0.001	134 +/- 25	141 +/- 26	< 0.001	139 +/- 27	143 +/- 27	< 0.001

n	*2108*	*611*		*2195*	*599*		*2625*	*770*	
Diastolic BP (mmHg)*	80 +/- 17	81 +/- 18	0.121	77 +/- 15	77 +/- 15	0.646	81 +/- 16	80 +/- 16	0.027
n	*2108*	*610*		*2189*	*596*		*2619*	*770*	
Killip class at entry			< 0.001			< 0.001			< 0.001
I	1749 (82.9)	441 (72.1)		1919 (88.8)	463 (77.4)		2381 (92.8)	622 (86)	
II	228 (10.8)	92 (15)		160 (7.4)	74 (12.4)		127 (5)	55 (7.6)	
III	111 (5.3)	70 (11.4)		64 (3)	48 (8)		45 (1.8)	39 (5.4)	
IV	21 (1)	9 (1.5)		19 (0.9)	13 (2.2)		12 (0.5)	7 (1)	
Max Killip class during hosp			< 0.001			< 0.001			< 0.001
I	1666 (79.8)	412 (69)		1861 (86.2)	441 (74.5)		2280 (90.7)	595 (84)	
II	239 (11.4)	91 (15.2)		173 (8)	79 (13.3)		135 (5.4)	57 (8.1)	
III	132 (6.3)	72 (12.1)		88 (4.1)	51 (8.6)		67 (2.7)	45 (6.4)	
IV	52 (2.5)	22 (3.7)		37 (1.7)	21 (3.5)		31 (1.2)	11 (1.6)	
STEMI or LBBB at entry	1215 (57.4)	294 (47.7)	< 0.001	1301 (57.7)	293 (47.3)	< 0.001	1488 (53.7)	332 (41.4)	< 0.001
**Biology at entry**									
Triglycerides** (mg/dl)	50 [36-72]	57 [41-82]	< 0.001	118 [85-168]	133 [97-179]	< 0.001	75 [48-118]	91 [57-147]	< 0.001
N	*1554*	*458*		*1727*	*475*		*2219*	*637*	
LDL cholesterol (mg/dl) *	135 +/- 44	126 +/- 50	< 0.001	124 +/- 47	104 +/- 42	< 0.001	133 +/- 51	115 +/- 54	< 0.001
N	*1401*	*408*		*1625*	*444*		*2115*	*594*	
HDL cholesterol (mg/dl)	49 +/- 17	45 +/- 14	< 0.001	47 +/- 16	43 +/- 16	< 0.001	47 +/- 19	43 +/- 17	< 0.001
n	*1421*	*416*		*1678*	*471*		*2190*	*620*	
Glycaemia (mg/dl) **	120 [100-140]	180 [140-250]	< 0.001	120 [100-140]	170 [140-240]	< 0.001	110 [100-130]	170 [130-220]	< 0.001
n	*1898*	*571*		*1991*	*555*		*2282*	*681*	
Haemoglobin (g/dl) *	13.9 +/- 1.8	13.6 +/- 2.0	< 0.001	14.2 +/- 1.8	13.7 +/- 1.9	< 0.001	14.2 +/- 1.8	13.7 +/- 2.0	< 0.001

n	*2048*	*594*		*2213*	*605*		*2665*	*774*	
CRP (UI/L) **	5.2 [3.0-16.0]	7.5 [4.0-27.0]	< 0.001	4.8 [2.0-10.0]	5.7 [3.0-17.5]	< 0.001	4 [2.3-11.0]	6 [3.0-16.0]	< 0.001
n	*1323*	*399*		*1745*	*496*		*2074*	*641*	
CKD-EPI Creatinine clearance (ml/min/1,73 m²)			< 0.001			< 0.001			< 0.001
≥ 90	527 (25.5)	110 (18.5)		787 (35.5)	159 (26.2)		1047 (39.4)	234 (30.6)	
60-89	856 (41.5)	222 (37.3)		1009 (45.5)	278 (45.8)		1162 (43.7)	299 (39.1)	
30-59	465 (22.5)	194 (32.6)		361 (16.3)	142 (23.4)		381 (14.3)	171 (22.4)	
15-29	57 (2.8)	30 (5.0)		38 (1.7)	19 (3.1)		30 (1.1)	33 (4.3)	
< 15 or dialysis	160 (7.8)	40 (6.7)		24 (1.1)	9 (1.5)		41 (1.5)	27 (3.5)	
HBA1c at entry (%)		6.9 [6.5-7.9]			7 [6.5-7.9]			6.9 [6.5-8.0]	
n		*355*			*405*			*546*	
**Coronarography - Reperfusion therapy**									
Coronary angiography performed	1869 (88.3)	530 (86.0)	0.134	2159 (95.8)	587 (94.7)	0.236	2723 (98.2)	770 (96.0)	< 0.001
CAD extension***			< 0.001			< 0.001			< 0.001
1 vessel	938 (52.9)	221 (43.5)		997 (49.2)	202 (35.9)		1231 (49.1)	252 (34.9)	
2-vessel	529 (29.8)	157 (30.9)		615 (30.3)	193 (34.3)		793 (31.6)	267 (37.0)	
3-vessel	308 (17.3)	130 (25.6)		416 (20.5)	168 (29.8)		483 (19.3)	203 (28.1)	
Any PCI attempt during initial hospitalization	1431 (67.6)	404 (65.6)	0.35	1793 (79.6)	460 (74.2)	0.004	2224 (80.3)	610 (76.2)	0,01
Any CABG during initial hospitalization	80 (3.8)	31 (5)	0.165	55 (2.4)	30 (4.8)	0.002	69 (2.5)	28 (3.5)	0,124
Initial LVEF (%) *	53.6 +/- 12.8	51.3 +/- 13.4	0.002	52.6 +/- 10.8	49.8 +/- 11.7	< 0.001	51.9 +/- 10.9	49.9 +/- 11.7	< 0.001
n	*1357*	*390*		*1924*	*522*		*2060*	*611*	
**Discharge**									

STEMI (discharge diagnosis)	1190 (56.2)	280 (45.5)	< 0.001	1308 (58.1)	292	< 0.001	1451 (52.4)	314 (39.2)	< 0.001
LVEF at discharge (%)			0.103			< 0.001			0.026
40 +	810 (79.6)	245 (75.4)		1370 (87.8)	334		1801 (84.6)	492 (80.8)	
< 40	207 (20.4)	80 (24.6)		191 (12.2)	87		329 (15.4)	117 (19.2)	
LVEF at discharge (%) *	52.7 +/- 12.8	50.9 +/- 13.3	0.037	53.4 +/- 10.1	51.1	< 0.001	52.2 +/- 10.3	50.9 +/- 10.7	0.007
n	*1017*	*325*		*1561*	*421*		*2130*	*609*	
GRACE score discharge*	112 +/- 34	120 +/- 30	< 0.001	107 +/- 31	118	< 0.001	107 +/- 30	117 +/- 29	< 0.001
n	*2040*	*590*		*2130*	*578*		*2456*	*704*	
Betablocker at discharge	1547 (73.1)	443 (71.9)	0.569	1579 (70.1)	452	0.167	2367 (85.4)	681 (84.9)	0.737
Statin at discharge	1670 (78.9)	480 (77.9)	0.608	1754 (77.8)	479	0.767	2553 (92.1)	726 (90.5)	0.153
ACEI/ARB at discharge	1262 (59.6)	410 (66.6)	0.002	1483 (65.8)	436	0.034	2032 (73.3)	615 (76.7)	0.055
Anti-platelet agent at discharge	1871 (88.4)	542 (88)	0.79	1951 (86.6)	544	0.44	2694 (97.2)	778 (97)	0.789

*: mean +/- SD
**: median [Interquartile range]

***: left main lesion classified as a 2-vessel disease

CAD: Coronary Artery Disease; PAD : Peripheral Arterial Disease; BP: Blood Pressure LDL: Low-density Lipoprotein; HDL: High-density Lipoprotein; CRP: C Reactive Protein; CKD-EPI: Chronic Kidney Disease – Epidemiology Collaboration; PCI: Percutaneous Coronary Intervention; CABG: Coronary Artery Bypass Grafting; LVEF: Left Ventricular Ejection Fraction; STEMI: ST Elevation Myocardial Infarction; ACEI: Angiotensin-Converting Enzyme Inhibitor; ARB: Angiotensin Receptor Blockers

Supplementary table 2: Table 2: Diabetes' treatment at admission and at discharge according to year of inclusion

	FAST-MI 2005 Patients With T2DM n=616		FAST-MI 2010 Patients With T2DM n=620		FAST-MI 2015 Patients with T2DM n=802	
	**n**	**%**	**n**	**%**	**n**	**%**
**At time of admission**						
Medical treatment of diabetes mellitus	396	64.3	380	61.3	459	57.2
Insulin treatment	53	8.6	63	10.2	106	13.2
Biguanide	229	37.2	259	41.8	325	40.5
Sulfonylurea	233	37.8	169	27.3	174	21.7
Alpha-glucosidase. Inhibitor	45	7.3	29	4.7	18	2.2
DPP4 inhibitor	0	0	45	7.3	116	14.5
Glucagon-Like Peptide-1 Receptor Agonists	0	0	3	0.5	16	2
Other oral antidiabetic agent	40	6.5	48	7.7	58	7.2
**Medical treatment of diabetes mellitus at discharge**						
Medical treatment	402	65.3	373	60.2	542	67.6
Insulin treatment	161	26.1	124	20	189	23.6
Biguanide	127	20.6	161	26	316	39.4
Sulfonylurea at discharge	183	29.7	129	20.8	170	21.2
Alpha-glucosidase. inhibitor	24	3.9	13	2.1	13	1.6
DPP4 inhibitor	0	0	45	7.3	130	16.2
Glucagon-Like Peptide-1 Receptor Agonists	0	0	3	0.5	14	1.7
Other oral antidiabetic agent	23	3.7	44	7.1	66	8.2

DPP4: Dipeptidyl Peptidase-4 ; GLP1: Glucagon Like Peptide 1

Supplementary table 3: Patients' characteristics associated with 1-year mortality

	Crude			Age-adjusted			Adjusted for age and registry		
	**OR**	**IC 95%**	**p**	**OR**	**IC 95%**	**p**	**OR**	**IC 95%**	**p**
**Demographic and social data**									
Male sex	0.75	0.62 - 0.90	0.002	1.47	1.21 - 1.80	< 0.001	1.50	1.22 - 1.83	< 0.001
Age (years)	1.09	1.08 - 1.09	< 0.001				1.09	1.08 - 1.09	< 0.001
**Cardiovascular risk factors**									
Hypertension	2.30	1.90 - 2.78	< 0.001	1.19	0.98 - 1.46	0.085	1.19	0.97 - 1.46	0.097
Dyslipidaemia	1.01	0.85 - 1.21	0.903	1.00	0.83 - 1.20	0.992	1.00	0.84 - 1.20	0.976
Smoking									
No	1.00	(ref)		1.00	(ref)		1.00	(ref)	
Former smoker	0.34	0.26 - 0.45	< 0.001	0.94	0.70 - 1.26	0.679	0.93	0.69 - 1.24	0.602
Active smoker	1.07	0.87 - 1.30	0.532	0.69	0.56 - 0.85	0.001	0.66	0.53 - 0.81	< 0.001
Diabetes	1.88	1.56 - 2.26	< 0.001	1.62	1.34 - 1.97	< 0.001	1.63	1.35 - 1.98	< 0.001
Diabetes									
No	1.00	(ref)		1.00	(ref)		1.00	(ref)	
Diet only	1.48	1.04 - 2.11	0.031	1.20	0.83 - 1.73	0.333	1.21	0.84 - 1.74	0.313
Treated (at discharge)	2.01	1.64 - 2.46	< 0.001	1.77	1.44 - 2.19	< 0.001	1.79	1.45 - 2.20	< 0.001
Diabetes									
No	1.00	(ref)		1.00	(ref)		1.00	(ref)	
Diet only	1.48	1.04 - 2.11	0.031	1.20	0.83 - 1.73	0.333	1.21	0.84 - 1.74	0.313
OAD agents at discharge	1.58	1.22 - 2.03	< 0.001	1.39	1.07 - 1.81	0.014	1.41	1.08 - 1.83	0.011
Insulin at discharge	2.93	2.22 - 3.86	< 0.001	2.60	1.94 - 3.47	< 0.001	2.59	1.93 - 3.45	< 0.001

Family history of CAD	0.36	0.27 - 0.47	< 0.001	0.63	0.47 - 0.84	0.002	0.63	0.47 - 0.84	0.002
**Past medical history**									
First cardiac event	0.36	0.31 - 0.44	< 0.001	0.51	0.43 - 0.62	< 0.001	0.52	0.43 - 0.62	< 0.001
Previous Heart failure	5.91	4.61 - 7.57	< 0.001	3.37	2.59 - 4.38	< 0.001	3.41	2.62 - 4.43	< 0.001
Previous PCI	1.78	1.44 - 2.19	< 0.001	1.49	1.20 - 1.85	< 0.001	1.57	1.26 - 1.95	< 0.001
Previous CABG	3.52	2.62 - 4.71	< 0.001	2.50	1.85 - 3.39	< 0.001	2.45	1.81 - 3.32	< 0.001
Previous (non-coronary) cardiac surgery	2.20	1.34 - 3.63	0.002	1.58	0.94 - 2.66	0.083	1.61	0.96 - 2.71	0.073
Previous AF or Flutter	3.15	2.26 - 4.38	< 0.001	1.62	1.15 - 2.28	0.006	1.61	1.14 - 2.26	0.007
Previous Stroke/TIA	2.98	2.28 - 3.89	< 0.001	1.81	1.37 - 2.39	< 0.001	1.79	1.35 - 2.36	< 0.001
PAD	3.97	3.16 - 4.98	< 0.001	2.82	2.22 - 3.57	< 0.001	2.75	2.17 - 3.49	< 0.001
Chronic renal failure	6.45	4.95 - 8.40	< 0.001	4.02	3.04 - 5.32	< 0.001	4.04	3.05 - 5.35	< 0.001
Dialysis	9.90	5.87 - 16.69	< 0.001	8.87	5.03 - 15.65	< 0.001	9.03	5.11- 15.95	< 0.001
Other life-threatening diseases	2.83	2.36 - 3.40	< 0.001	2.08	1.72 - 2.52	< 0.001	2.36	1.94 - 2.87	< 0.001
**Initial presentation**									
Pre-hospitalization heart failure	4.71	3.81 - 5.83	< 0.001	2.78	2.22 - 3.48	< 0.001	2.66	2.13 - 3.34	< 0.001
Syncope	1.64	1.14 - 2.38	0.008	1.47	1.001 - 2.17	0.049	1.44	0.98 - 2.12	0.064
Pre-hospital cardiac arrest	0.90	0.39 - 2.06	0.802	1.34	0.57 - 3.15	0.496	1.31	0.56 - 3.08	0.539
Typical chest pain	0.49	0.41 - 0.60	< 0.001	0.65	0.53 - 0.80	< 0.001	0.66	0.54 - 0.81	< 0.001
**Clinical characteristics at admission**									
BMI (kg/m^2)	0.93	0.91 - 0.95	< 0.001	0.97	0.94 - 0.99	0.004	0.97	0.95 - 0.99	0.005
Obesity (BMI ≥ 30 kg/m^2)	0.63	0.49 - 0.82	< 0.001	0.86	0.66 - 1.13	0.279	0.87	0.66 - 1.14	0.304
Heart rate (for 10 bpm)	1.15	1.11 - 1.20	< 0.001	1.13	1.08 - 1.18	< 0.001	1.13	1.08 - 1.17	< 0.001
Systolic BP (for 10 mmHg)	0.94	0.90 - 0.97	< 0.001	0.90	0.87 - 0.93	< 0.001	0.90	0.87 - 0.93	< 0.001

Diastolic BP (for 10 mmHg)	0.80	0.75 - 0.84	< 0.001	0.87	0.82 - 0.93	< 0.001	0.87	0.82 - 0.93	< 0.001
Killip class at entry									
I	1.00	(ref)		1.00	(ref)		1.00	(ref)	
II	3.97	3.14 - 5.02	< 0.001	2.45	1.91 - 3.13	< 0.001	2.37	1.85 - 3.03	< 0.001
III	7.18	5.51 - 9.36	< 0.001	3.97	3.00 - 5.24	< 0.001	3.79	2.86 - 5.03	< 0.001
IV	5.81	3.32 - 10.15	< 0.001	4.60	2.54 - 8.32	< 0.001	4.45	2.45 - 8.09	< 0.001
Max Killip class during hosp									
I	1.00	(ref)		1.00	(ref)		1.00	(ref)	
II	4.23	3.35 - 5.33	< 0.001	2.52	1.98 - 3.22	< 0.001	2.43	1.90 - 3.11	< 0.001
III	5.76	4.43 - 7.50	< 0.001	3.22	2.45 - 4.25	< 0.001	3.08	2.33 - 4.07	< 0.001
IV	4.95	3.26 - 7.50	< 0.001	4.10	2.65 - 6.35	< 0.001	3.94	2.54- 6.11	< 0.001
STEMI or LBBB at entry	0.66	0.55 - 0.78	< 0.001	0.87	0.72 - 1.04	0.125	0.86	0.71 - 1.03	0.096
Biology at admission									
Triglycerides									
Tertile 1	1.00	(ref)		1.00	(ref)		1.00	(ref)	
Tertile 2	0.85	0.66 - 1.08	0.184	1.00	0.78 - 1.29	0.999	1.12	0.85 - 1.47	0.427
Tertile 3	0.52	0.39 - 0.69	< 0.001	0.82	0.62 - 1.10	0.191	0.98	0.70 - 1.38	0.928
HDL cholesterol (for 100 mg/dl)	0.97	0.50 - 1.87	0.918	0.35	0.16 - 0.75	0.007	0.33	0.15 - 0.72	0.005
LDL cholesterol (for 100 mg/dl)	0.39	0.30 - 0.51	< 0.001	0.58	0.44 - 0.77	< 0.001	0.55	0.41 - 0.74	< 0.001
Glycaemia									
Tertile 1	1.00	(ref)		1.00	(ref)		1.00	(ref)	
Tertile 2	1.41	1.09 -1.83	0.009	1.28	0.98 - 1.67	0.071	1.26	0.96 - 1.65	0.092
Tertile 3	2.25	1.77 - 2.86	< 0.001	1.80	1.40 - 2.30	< 0.001	1.74	1.36 - 2.24	< 0.001
Haemoglobin (for 1 g/dl)	0.65	0.62 - 0.68	< 0.001	0.74	0.70 - 0.77	< 0.001	0.74	0.70 - 0.77	< 0.001
CRP									

Tertile 1	1.00	(ref)		1.00	(ref)		1.00	(ref)	
Tertile 2	1.62	1.14- 2.30	0.007	1.61	1.13- 2.30	0.008	1.58	1.11 - 2.26	0.011
Tertile 3	5.23	3.87 - 7.08	< 0.001	4.23	3.10 - 5.75	< 0.001	4.15	3.04 - 5.66	< 0.001
CKD-EPI Creatinine clearance (ml/min/1,73 m²)									
≥ 90	1.00	(ref)		1.00	(ref)		1.00	(ref)	
60-89	1.88	1.37 - 2.58	< 0.001	0.73	0.52 - 1.04	0.081	0.71	0.50 - 1.01	0.057
30-59	7.68	5.68 - 10.38	< 0.001	1.78	1.24 - 2.57	0.002	1.69	1.17 - 2.44	0.006
15-29	19.41	12.94 - 29.10	< 0.001	3.94	2.47 - 6.29	< 0.001	3.71	2.32 - 5.95	< 0.001
< 15 or dialysis	9.93	6.62 - 14.90	< 0.001	3.60	2.31 - 5.61	< 0.001	3.32	2.11 - 5.24	< 0.001
**Coronarography - Reperfusion therapy**									
Disease extension *									
1 vessel	1.00	(ref)		1.00	(ref)		1.00	(ref)	
2-vessel	1.50	1.35 - 1.67	< 0.001	1.21	1.08 - 1.34	0.001	1.21	1.09 - 1.35	< 0,001
3-vessel	2.30	2.06 - 2.56	< 0.001	1.59	1.42 - 1.77	< 0.001	1.60	1.43 - 1.78	< 0,001
Any PCI attempt during initial hospitalization	0.27	0.23 - 0.33	< 0.001	0.41	0.34 - 0.50	< 0.001	0.43	0.36 - 0.52	< 0.001
Any CABG during initial hospitalization	0.62	0.34 - 1.14	0.124	0.65	0.35 - 1.20	0.169	0.63	0.34 -1.16	0.14
Initial LVEF (for 1% increase)	0.95	0.94 - 0.96	< 0.001	0.96	0.95 - 0.97	< 0.001	0.96	0.95 - 0.97	< 0.001
**Discharge**									
STEMI (discharge diagnosis)	0.60	0.50 - 0.71	< 0.001	0.81	0.67 - 0.97	0.024	0.80	0.67 - 0.96	0.018
LVEF at discharge < 40%	3.55	2.81 - 4.48	< 0.001	2.76	2.16 - 3.51	< 0.001	2.68	2.10 - 3.41	< 0.001
LVEF at discharge (for 1% increase)	0.94	0.94 - 0.95	< 0.001	0.95	0.95 - 0.96	< 0.001	0.96	0.95 - 0.97	< 0.001
GRACE score discharge (for 10 points increase)	1.51	1.46 - 1.56	< 0.001	1.50	1.42 - 1.58	< 0.001	1.49	1.41 - 1.57	< 0.001

Betablocker at discharge	0.62	0.51 - 0.75	< 0.001	0.73	0.60 - 0.89	0.001	0.77	0.63 - 0.94	0.011
Statin at discharge	0.49	0.40 - 0.60	< 0.001	0.66	0.54- 0.81	< 0.001	0.70	0.57 - 0.87	0.001
ACEI/ARB at discharge	0.63	0.53 - 0.75	< 0.001	0.74	0.61 - 0.89	0.001	0.77	0.63 - 0.92	0.005
Anti-platelet agent at discharge	0.75	0.56 - 0.99	0.039	0.89	0.67 - 1.19	0.437	0.96	0.71 - 1.28	0.774

*: In patients with abnormal coronary angiography. Left main lesion classified as a 2-vessel disease

CAD: Coronary Artery Disease; PAD: Peripheral Arterial Disease; BP: Blood Pressure LDL: Low-density Lipoprotein; HDL: High-density Lipoprotein; CRP: C Reactive Protein; CKD-EPI: Chronic Kidney Disease – Epidemiology Collaboration; PCI: Percutaneous Coronary Intervention; CABG: Coronary Artery Bypass Grafting; LVEF: Left Ventricular Ejection Fraction; STEMI: ST Elevation Myocardial Infarction; ACEI: Angiotensin-Converting Enzyme Inhibitor; ARB: Angiotensin Receptor Blockers

Supplementary table 4: Model of one-year mortality prediction developed for the propensity score matching (Logistic regression model)

	OR	IC 95%	p
**Male gender**	1.57	1.24 - 2.00	< 0.001
**Age (years)**	1.05	1.04 - 1.06	< 0.001
**Previous coronary event (MI, PCI, or CABG)**	1.43	1.14 - 1.78	0.002
**Heart failure**	1.84	1.35 - 2.51	< 0.001
**PAD**	1.44	1.08 - 1.93	0.012
**Other life-threatening diseases**	1.65	1.32 - 2.05	< 0.001
**BMI (kg/m²)**	0.97	0.95- 0.99	0.038
**Heart rate at entry (bpm)**			
< 70	1.00	(ref)	
≥ 70	1.48	1.16 - 1.90	0.002
Unknown	0.87	0.30 - 2.54	0.801
**Systolic BP at entry (mmHg)**			
< 130	1.00	(ref)	
≥ 130	0.67	0.54 - 0.83	< 0.001
Unknown	0.96	0.33 - 2.78	0.944
**Max Killip class during hospitalization**			
I - II	1.00	(ref)	
III - IV	1.45	1.08 - 1.94	0.012
Unknown	0.87	0.54 - 1.39	0.558
**Haemoglobin (g/dl)**	0.65	0.62 - 0.68	< 0.001
< 13.5	1.00	(ref)	
≥ 13.5	0.48	0.38 - 0.61	< 0.001
Unknown	0.46	0.22 - 0.93	0.031
**CRP (UI/L)**			
< 10	1.00	(ref)	
≥ 10	2.14	1.66 - 2.77	< 0.001
Unknown	1.75	1.33 - 2.30	< 0.001
**CKD-EPI creatinine clearance (ml/min/1.73m²)**			
≥ 30	1.00	(ref)	
< 30 or dialysis	1.93	1.43 - 2.61	< 0.001
Unknown	0.81	0.37 - 1.77	0.597
**Coronarography**			

1 vessel	1.00	(ref)	
2-vessel	1.19	0.88 - 1.61	0.253
3-vessel	1.67	1.24 - 2.25	0.001
Unknown	1.45	1.00 - 2.11	0.053
**Any PCI attempt during initial hospitalization**	0.59	0.44 - 0.79	< 0.001
**CABG during initial hospitalization**	0.45	0.23 - 0.89	0.021
**LVEF at discharge (%)**			
≥ 40	1.00	(ref)	< 0.001
< 40	1.88	1.42 - 2.49	< 0.001
Unknown	1.29	1.01 - 1.63	0.038

MI: Myocardial Infarction; PCI: Percutaneous Coronary Intervention; CABG: Coronary Artery Bypass Grafting; PAD Peripheral Arterial Disease; BMI Body Mass Index; BP Blood Pressure; CRP: C-Reactive Protein; CKD-EPI: Chronic Kidney Disease Epidemiology Collaboration; LVEF: Left Ventricular Ejection Fraction

Supplementary figures' legends

Supplementary figure 1: Comparison of mortality between patients with and patients without T2DM according to the type of ACS in propensity matched population (Panel A: Non-ST elevation Acute coronary syndrome, Panel B: ST elevation Acute coronary syndrome)

Supplementary Figure 1 caption: Comparison of survival in patients with and patients without T2DM, in propensity matched population stratified according to the type of acute coronary syndrome. $HR_{Diabetes}$ for non-STEMI=1.34 [1.16 - 1.55] p<0.001, $HR_{Diabetes}$ for STEMI=1.17 [0.99 - 1.39] p=0.067.

Supplementary figure 2: Comparison of mortality between patients with and patients without T2DM according to the registry of inclusion, in propensity matched population (Panel A: FAST MI 2005; Panel B: FAST MI 2010, and Panel C: FAST MI 2015).

Supplementary Figure 2 caption: Comparison of survival in patients with and patients without T2DM, stratified according to the registry of inclusion, in propensity matched population. $HR_{Diabetes}$ for FAST-MI 2005 = 1.09 [0.92 - 1.30] p=0.303, $HR_{Diabetes}$ for FAST-MI 2010 = 1.37 [1.14 - 1.65] p=0.001, $HR_{Diabetes}$ for FAST-MI 2015 = HR=1.66 [1.31 – 2.10] p<0.001.

Supplementary Figure 1:

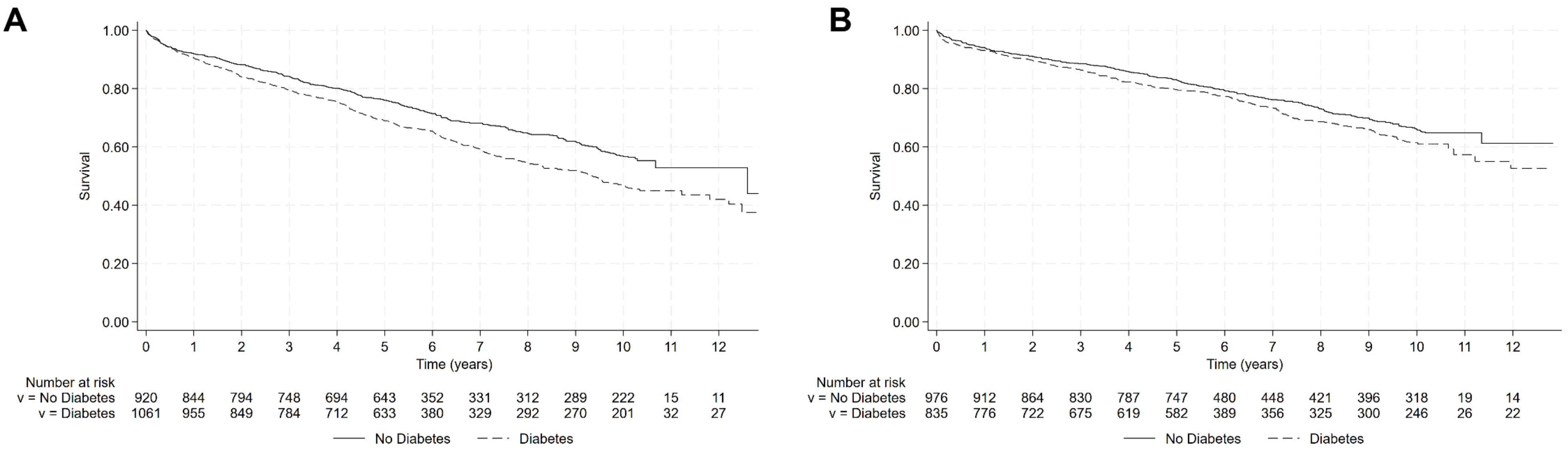

Supplementary Figure 2:

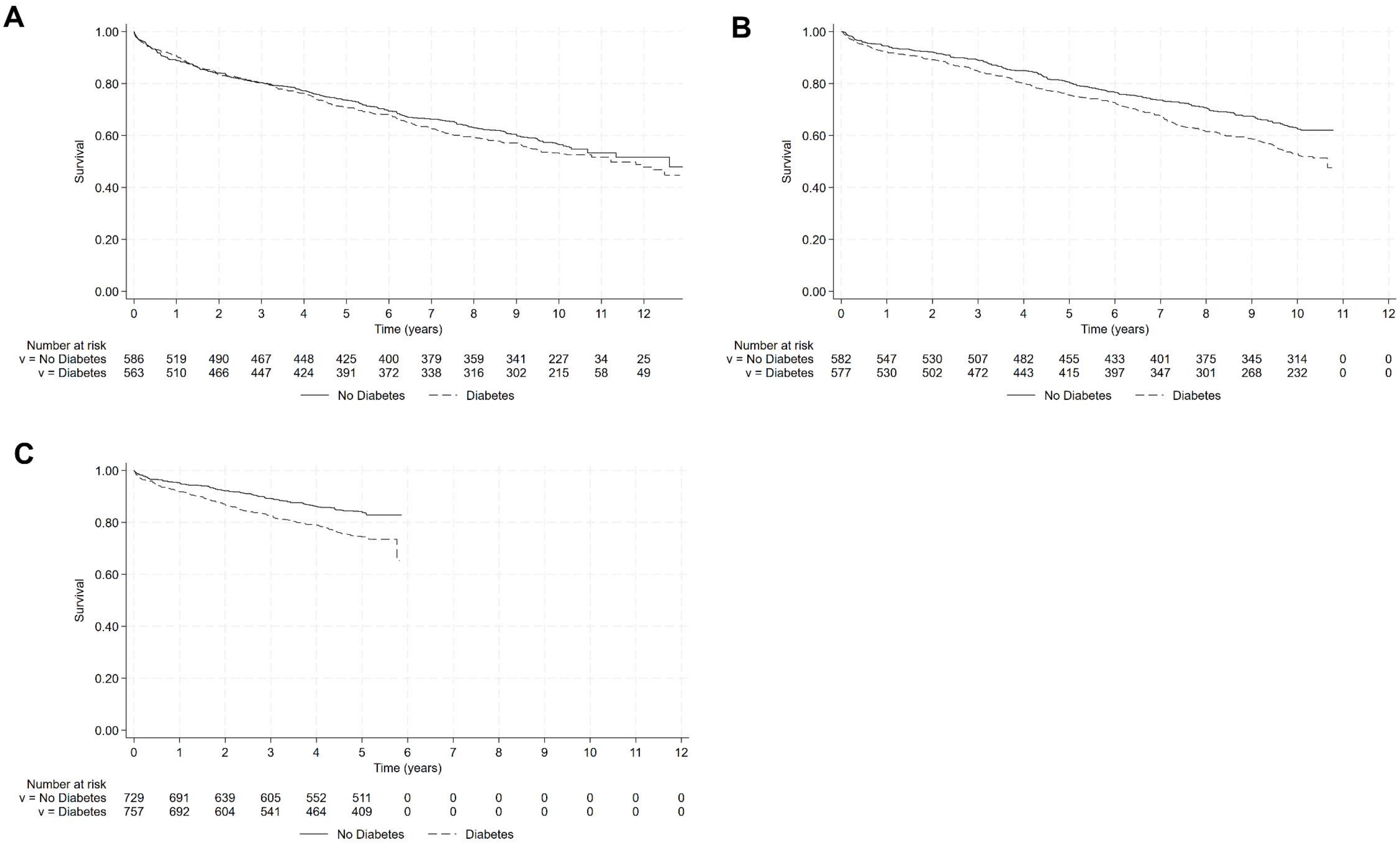

Supplement: Supplementary file 1 [file Datasheet1.pdf]
